# Regulation of Translation by TOR, eIF4E and eIF2α in Plants: Current Knowledge, Challenges and Future Perspectives

**DOI:** 10.3389/fpls.2017.00644

**Published:** 2017-04-26

**Authors:** Ane Sesma, Carmen Castresana, M. Mar Castellano

**Affiliations:** ^1^Centro de Biotecnología y Genómica de Plantas, Universidad Politécnica de Madrid – Instituto Nacional de Investigación y Tecnología Agraria y AlimentariaMadrid, Spain; ^2^Departamento Biotecnología y Biología Vegetal, Universidad Politécnica de MadridMadrid, Spain; ^3^Centro Nacional de Biotecnología – Consejo Superior de Investigaciones Científicas (CSIC)Madrid, Spain

**Keywords:** translation initiation, stress, plant development, TOR, eIF2α, eIF4E

## Abstract

An important step in eukaryotic gene expression is the synthesis of proteins from mRNA, a process classically divided into three stages, initiation, elongation, and termination. Translation is a precisely regulated and conserved process in eukaryotes. The presence of plant-specific translation initiation factors and the lack of well-known translational regulatory pathways in this kingdom nonetheless indicate how a globally conserved process can diversify among organisms. The control of protein translation is a central aspect of plant development and adaptation to environmental stress, but the mechanisms are still poorly understood. Here we discuss current knowledge of the principal mechanisms that regulate translation initiation in plants, with special attention to the singularities of this eukaryotic kingdom. In addition, we highlight the major recent breakthroughs in the field and the main challenges to address in the coming years.

## Introduction

In eukaryotes, canonical cap-dependent translation begins with eIF4E recognition of the cap structure (7-methyl guanosine) at the 5′-end of the mRNA and formation of the eIF4F complex. Within this complex, eIF4G interacts with several factors, allowing mRNA recircularization and recruitment of the preinitiation complex 43S (PIC) to the mRNA. Once loaded, this complex, which consists of the small ribosomal subunit 40S, the ternary complex eIF2/GTP/tRNA_i_^met^ and the factors eIF3, eIF1, and eIF1A, scans the mRNA in the 5′-3′ direction until an initiation codon is found. At that point, the ribosomal subunit 60S is loaded and the elongation phase begins (Jackson et al., 2010; Hinnebusch et al., 2016).

Regulation of protein synthesis is a widespread, dynamic mechanism that controls gene expression in eukaryotes. This regulation takes place mainly, but not exclusively, during the translation initiation phase and involves the regulation of the activity of the master kinase target of rapamycin (TOR) and two important translation initiation factors, eIF4E and eIF2α (Chu et al., 2013; Hinnebusch et al., 2016). Although regulation of these three main players has been studied profusely in other eukaryotes, the information available as to how these proteins regulate translation in plants is very limited. This review focuses on specific aspects of their involvement in translation initiation in plants, introducing what is known in other organisms, what we know about their regulation in plants, and how this regulation impinges on specific aspects of plant development and response to environmental cues.

## The TOR Signaling Cascade

The TOR protein kinase is a central regulator of growth in response to nutrients in eukaryotic cells. The importance of this signaling pathway is shown by the large number of papers published annually (see Dobrenel et al., 2016a; Eltschinger and Loewith, 2016; Gonzalez and Hall, 2017 for recent reviews), and the celebration of the 25-year anniversary of its discovery (Hall, 2016). The two yeast TOR kinase genes were first identified during a screen designed to seek the targets of the antiproliferative drug rapamycin (Heitman et al., 1991). Soon after, it was observed that translation initiation was altered in yeast TOR mutants (Barbet et al., 1996). It is now well established that TOR integrates the signals that perceive the nutritional status of the cell and regulates downstream processes essential for proliferation and growth. These include the ability to modulate translation initiation (Ma and Blenis, 2009; Thoreen et al., 2012; Nandagopal and Roux, 2015), maintenance of lysosome identity (Yu et al., 2010; Munson et al., 2015), autophagy (Noda and Ohsumi, 1998), and synthesis of ribosomes and tRNAs (Ma and Blenis, 2009). Whereas yeast has two TOR proteins, some filamentous fungi, animals, and plants have only one (Franceschetti et al., 2011; Dobrenel et al., 2016a; Eltschinger and Loewith, 2016). Yeast and mammalian TOR proteins form two widely conserved multiprotein complexes that differ structurally and functionally, TORC1 and TORC2; only TORC1 is rapamycin-sensitive (Eltschinger and Loewith, 2016). The ability of these TORC complexes to interact with specific protein partners controls the diverse downstream outputs of the TOR cascade.

In plants, analysis of the TOR pathway has been a challenge because of the embryo lethality of knockout TOR mutants (Menand et al., 2002), and the less reliable rapamycin sensitivity of these eukaryotes (Rexin et al., 2015; Dobrenel et al., 2016a). The latter is due to the differences in amino acid residues in the 12 kDa FK506-binding proteins (FKBP12) of plants. The generation of TOR inducible mutants and silenced lines (Deprost et al., 2007; Caldana et al., 2013; Dobrenel et al., 2016b), the introduction of the yeast FKBP12 in Arabidopsis, which increases rapamycin sensitivity (Sormani et al., 2007), together with newly developed drugs that target TOR such as Torin and AZD-8055, have provided tools to dissect the role of TOR in plants. Although all TORC1 components are present, no clear orthologs of the TORC2 subunits AVO1 and AVO3 have yet been found in plants (Robaglia et al., 2012; Maegawa et al., 2015).

## TORC1 and the Control of Translation Initiation

In yeast and animals, TORC1 activation by nutrient signals coordinately controls various components of the translation initiation machinery by direct or indirect phosphorylation of a subset of proteins (Ma and Blenis, 2009). These include the translation initiation factors eIF4G, eIF4B, 4E-BPs, and the 40S ribosomal S6 kinases (S6K1 and S6K2). Furthermore, TORC1 can control general protein synthesis and the selective translation of specific mRNAs, including those with 5′ terminal oligopyrimidine (TOP) tracts (Thoreen et al., 2012). These TOP mRNAs encode ribosomal and other proteins that control translation. Although the mechanism by which these mTOR-dependent mRNAs are selected is not yet clear, several features of their 5′ UTR has allowed their classification in two functional subsets of transcripts whose translation initiation is regulated differently (Gandin et al., 2016). Not only nutrient starvation, but also other stresses can modulate the TOR cascade and canonical translation initiation, as shown during hypoxia, when mTOR inactivation reduces the translation of several TOP mRNAs and overall protein biosynthesis (Spriggs et al., 2010).

Plant lines in which TOR is silenced have reduced polysomal content (Deprost et al., 2007), an observation that supports TOR involvement in translation regulation. TOR also reduces the transcription and translation rates of nuclear genes that encode plastidic ribosomal proteins, suggesting protein synthesis defects in chloroplasts. This correlates with the chlorotic phenotype observed in *TOR*-silenced plants (Dobrenel et al., 2016b).

Arabidopsis S6K conserves the main regulatory phosphorylation sites found in human S6K (Dobrenel et al., 2016a), and phosphorylation of S6K1 has been used to monitor TORC1 activity in plants (Xiong and Sheen, 2011). This is an important TOR effect, since the S6K pathway not only stimulates overall protein synthesis but also eIF3h-mediated translation reinitiation after an upstream open reading frame (uORF) (Schepetilnikov et al., 2013), a frequent feature found in plant mRNAs (von Arnim et al., 2014). Auxin treatment activates TOR in Arabidopsis seedlings, and stimulates TOR association with polysomes, where S6K1 is phosphorylated (Bogre et al., 2013; Schepetilnikov et al., 2013). The loading of the translation initiation factor eIF3h into polysomes in response to auxin is impaired in TOR-deficient mutants with an inactive S6K form, suggesting that eIF3h is possibly phosphorylated by the TOR/S6K1 pathway (Schepetilnikov et al., 2013). With respect to environmental pressures, TOR activity can modulate the plant response to osmotic stress through the S6K1 kinase pathway (Mahfouz et al., 2006; Deprost et al., 2007). Other evidence pointing to TOR participation in plant adaptation derives from the induction of the two S6K gene homologs in Arabidopsis by cold and salinity (Mizoguchi et al., 1995). Although the S6K pathway is conserved in plants, clear orthologs of the other main target of TOR, the eIF4E-binding proteins (4E-BPs), have not been identified in this kingdom (see below for details).

## Regulation of eIF4E Activity in Animals by its Association to Different Proteins

In animals, eIF4E translational activity is tightly regulated by a myriad of proteins that regulate eIF4E function by phosphorylation (Waskiewicz et al., 1997) or by binding directly to eIF4E. These latter proteins, which are one of the focus of this review, modulate general and specific translation.

Probably the best-known eIF4E translational regulators are the mammalian 4E-BPs (Lin et al., 1994; Pause et al., 1994; Poulin et al., 1998). These proteins interact with eIF4E through multiple contacts to the lateral and dorsal surface of eIF4E (Paku et al., 2012; Lukhele et al., 2013; Peter et al., 2015). The dorsal interaction comprises the so-called 4E-binding motif (4E-BM), a canonical sequence YXXXXLØ (where Ø denotes a hydrophobic amino acid) present in 4E-BPs. Since the same motif is used by eIF4G for eIF4E binding (Mader et al., 1995; Marcotrigiano et al., 1999), the output of 4E-BPs/eIF4E interaction is the displacement of eIF4G from the eIF4E-eIF4G complex, which leads to general inhibition of mRNA translation (Haghighat et al., 1995). 4E-BPs interaction with eIF4E is intimately coupled to their phosphorylation status, which is controlled and adapted to physiological conditions through the master kinase TOR (Nandagopal and Roux, 2015). After TOR phosphorylation, mammalian 4E-BPs dissociate from eIF4E, whereas in their hypophosphorylated state, 4E-BPs form a tight complex with eIF4E (Pause et al., 1994).

Along with the 4E-BPs, other proteins known as 4E-interacting partners associate to eIF4E through canonical 4E-BM or similar structures (Napoli et al., 2008). In general terms, these proteins support multiple protein–protein interactions that create bridges between the 5′ and 3′ UTR of specific mRNAs, rendering them inactive for translation (Wells, 2006; Rhoads, 2009). Although these eIF4E interactors control specific animal developmental programs, such a mechanism has not been found yet in plants.

In addition to these translational regulators, several eIF4E interactors were recently implicated in eIF4E-dependent mRNA export and degradation (Nishimura et al., 2015; Osborne and Borden, 2015). Some of these proteins, such as LRPPRC, PRH, or 4E-T, interact with eIF4E through the canonical 4E-BM (Dostie et al., 2000; Topisirovic et al., 2003, 2009), which highlights the importance of this domain in eIF4E binding and regulation.

## Regulation of eIF4E Activity by its Association to Different Proteins in Plants

As described above, the most common and powerful tool for regulation of eIF4E activity in animals is protein association to the dorsal surface of eIF4E. For this reason, it is surprising that despite the conservation of the amino acids involved in the eIF4E/eIF4G interaction and the precise regulation of translation in different developmental and environmental conditions, no clear homologs of these eIF4E regulators have yet been found in plants. This is especially surprising for 4E-BPs, which appear to have been conserved throughout the evolution of many eukaryotic species, but specifically lost in plants (Hernandez et al., 2010).

Besides the lack of plant orthologs for the 4E-BPs and eIF4E-interacting partners, the existence of proteins that regulate eIF4E activity through eIF4E association remains an open question. Different studies reported the identification of proteins bearing the consensus 4E-BM that bind eIF4E and eIFiso4E (Freire et al., 2000; Freire, 2005; Lázaro-Mixteco and Dinkova, 2012), although their role in translation has yet to be elucidated. Apart from these proteins, an Arabidopsis database search retrieves more than 6900 proteins that contain one or more canonical eIF4E-binding domains (YXXXXLØ) (Toribio et al., 2016), that therefore might bind eIF4E and regulate its function. The number of possible plant eIF4E interactors could be larger if we consider that the canonical domain can have variations at the 3′ end and that some structures like the reversed L-shaped motif can also promote eIF4E binding (Napoli et al., 2008). Other evidence that supports the existence of these eIF4E regulators are the presence of conserved RNA-binding proteins in plants as the case of Brn, which mediates eIF4E translational inhibition of targeted mRNAs in animals (Kim et al., 2013).

It is worth to mention that wheat eIF4E and eIFiso4E show different isoelectric isoforms that are compatible with changes in their phosphorylation state (Gallie et al., 1997). Although the kinases involved have not been identified, the existence of different isoelectric states opens the possibility that these modifications could regulate translation initiation during plant development and/or in response to environmental cues.

## Translation Regulation by the Initiation Factor eIF2α

Inhibition of canonical translation by eIF2α phosphorylation has been analyzed exhaustively (Hinnebusch et al., 2016). Studies in yeast and other eukaryotes showed the eIF2 function in formation of the ternary complex Met-tRNA_i_^Met^-eIF2-GTP, needed to couple the initiating Met-tRNA_i_^Met^ at the first AUG in the 5′ leader of mRNAs. The resulting eIF2-GDP complex is recycled by eIF2B to eIF2-GTP, which binds a new molecule of Met-tRNA_i_^Met^ and forms a new ternary complex to initiate translation. eIF2, one of the best-characterized translation initiation factors, is composed of three subunits, eIF2α, eIF2β, and eIF2γ. Phosphorylation of the conserved Ser51 residue in the eIF2α subunit inhibits eIF2B dissociation from the eIF2-GDP complex and thus, formation of a new ternary complex, whose depletion arrests initiation of protein synthesis. Phosphorylation of eIF2α is a key mechanism that controls mRNA translation in eukaryotes in response to stress. In yeast, the general control non-derepressible 2 (GCN2) kinase phosphorylates eIF2α during nutrient starvation. GCN2 is part of a complex also comprised of GCN1 and GCN20 proteins, necessary to trigger eIF2α phosphorylation (Hinnebusch, 2005; Castilho et al., 2014). In mammals, protein kinases in addition to GCN2 phosphorylate eIF2α in various stress conditions including nutrient starvation, protein misfolding, or immune responses (Harding and Ron, 2002; Baker et al., 2012; Donnelly et al., 2013).

In Arabidopsis, GCN2 mediates eIF2α phosphorylation after stress treatments such as UV light, amino acid starvation, cadmium, oxidative stress, and wounding, and it is so far the only eIF2α kinase identified in plants (Lageix et al., 2008; Zhang et al., 2008; Sormani et al., 2011; Wang et al., 2016). In addition, ILITHYIA (ILA), the Arabidopsis homolog of yeast GCN1, is needed to promote eIF2α phosphorylation in response to cold (Wang et al., 2016). Despite this evidence, the functional relevance of this regulatory pathway in plant adaptation to stress is not yet completely understood (see Echevarria-Zomeño et al., 2013; Browning and Bailey-Serres, 2015 for recent reviews). In plants, GCN2-dependent eIF2α phosphorylation is reported to regulate protein synthesis, although this mechanism as a general inhibitor of translation is limited to the responses to the purine synthesis inhibitor 8-azaadenine and the amino acid synthesis inhibitor chlorsulfuron (Lageix et al., 2008). Moreover, data are contradictory regarding the role of GCN2 in plant adaptation to amino acid deprivation (Zhang et al., 2008; Faus et al., 2015).

In addition to abiotic and nutritional stresses, recent evidence suggests a function for GCN2 and eIF2α phosphorylation in plant immunity, although their role remains elusive. eIF2α phosphorylation is reported in response to *Pseudomonas syringae* pv. *maculicola* ES4326/avrRpt2 infection (Pajerowska-Mukhtar et al., 2012), but its influence on bacterial growth has yet to be determined. Adult *gcn2* plants show enhanced resistance to the necrotroph *Pectobacterium carotovorum* subsp. *carotovorum* and the biotrophic fungus *Golovinomyces cichoracearum*, a response that contrasts with the enhanced susceptibility of young *gcn2* plants to *G. cichoracearum* or *Hyaloperonospora arabidopsidis* inoculation (Liu et al., 2015). Other studies reported activation of eIF2α phosphorylation in response to treatment with the defense-related hormones salicylic acid, jasmonic acid, the ethylene precursor ACC, and the priming agent β-aminobutyric acid (Lageix et al., 2008; Luna et al., 2014; Wang et al., 2016).

## Challenges and Future Perspectives

The recent development of techniques for obtaining ribosome footprints in plants, by direct isolation of monosomes (Ribo-seq) (Merchante et al., 2015, 2016; Hsu et al., 2016) or by TRAP-SEQ (translating ribosome affinity purification-RNA sequencing) (Wang and Jiao, 2014; Juntawong et al., 2015; Reynoso et al., 2015), have revolutionized translation studies; they allow determination of exact ribosome positions on a genome-wide scale at single-codon resolution. These techniques have already been used to identify global features in translating mRNAs (Hu et al., 2016; Zhao et al., 2016), translating mRNAs in chloroplasts (Zoschke et al., 2013; Chotewutmontri and Barkan, 2016) and mRNAs regulated at the translational level during developmental processes such as seed germination and in response to stress conditions or plant hormones (Mustroph et al., 2009; Juntawong et al., 2014; Merchante et al., 2015; Bai et al., 2016). In addition, the incorporation of a non-canonical aminoacid, azidohomoalanine (AHA), has recently been used to monitor newly synthesized proteins in plants. The use of AHA was firstly reported by Echevarria-Zomeño et al. (2015), where AHA was described to mark *de novo* synthesized HSP90 and HSP70 proteins under heat stress conditions in Arabidopsis. This method, coupled to tandem liquid chromatography-mass spectrometry (LC-MS), has now been implemented to allow non-radioactive analysis of protein synthesis in plants (Glenn et al., 2017). All these techniques will be extremely helpful for identifying and characterizing the mechanisms that regulate translation in response to nutritional and environmental cues.

The recent development of chemical genetic tools and cellular assays for analysis of TOR pathway in plants will help to identify new targets of this pathway and to understand its involvement in translation regulation. It will also be relevant to clarify the regulatory activity of TOR on TOP mRNAs, as well as its role in regulating plant adaptation through selective translation of ribosomal proteins. In addition to translation, glucose-mediated TOR signaling has been found to play an important role at transcriptional level in Arabidopsis (Xiong et al., 2013).

Since the function of putative plant eIF4E interactors has not been studied in detail, considerable effort is needed to determine the role of these proteins in mRNA translation, export or decay, and how such regulation could affect plant development or responses to environmental stimuli.

An exclusive feature of plant translational machinery is the presence along with eIF4E of eIFiso4E isoforms, which mediate the translation of specific mRNA populations as part of the eIFiso4F complexes (Mayberry et al., 2009; Martinez-Silva et al., 2012; Chen et al., 2014). Therefore, it would be of interest to analyze the possible specialization of the eIF4E putative regulators in the selective regulation of eIF4E and eIFiso4E proteins. It will also be important to study the nature of eIF4E and eIFiso4E post-translational modifications and their function in the control of translation initiation in plants.

The effort to define the role of phosphorylated eIF2α during plant adaptation to environmental changes highlights current interest in this area. Reports with contrasting results nonetheless emphasize the need for additional studies to clarify the participation in plant immunity of eIF2α phosphorylation and of the TOR pathway. As inhibition of translation mediated by eIF2α phosphorylation is less severe in plants than in mammals, it is necessary to clarify its role in plant adaptation to stress; identification of plant mRNAs targeted by this regulatory mechanism would constitute a major breakthrough.

**FIGURE 1:**
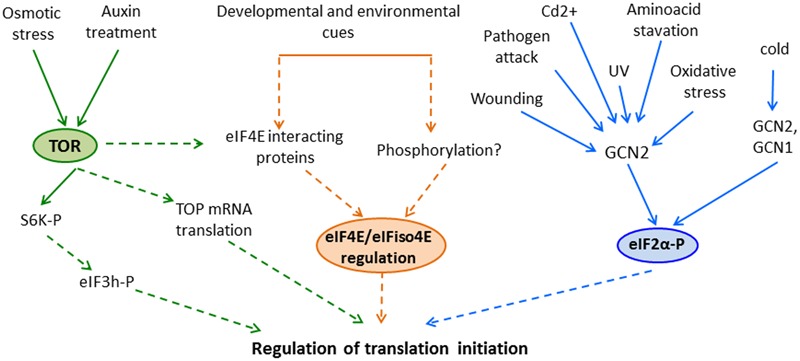
**Regulation of translation initiation by the (TOR) pathway, eIF4E activity and eIF2α phosphorylation in response to developmental and environmental cues in plants.** Different treatments activate plants’ TOR and GCN2 that promote downstream phosphorylation of S6K and eIF2α, respectively. In addition, eIF4E and eIFiso4E activity could be also regulated in these organisms, although the possible mechanisms involved in this regulation has not been elucidated yet. Despite that these events could lead to regulation of translation initiation (based on the information in other eukaryotes), in some cases the precise role of these pathways in translational control remains unclear in plants. Solid lines highlight experimentally demonstrated associations among processes; in contrast, dashed lines represent possible links that are missing or unresolved in plants.

In this review, we have focused on the regulation of the TOR pathway and eIF4E and eIF2α translation initiation factors by developmental and environmental cues (**Figure 1**). Nevertheless, when analyzing translation regulation during plant response to environmental changes, other mechanisms including those that affect translation elongation and termination, or formation of cytoplasmic ribonucleoprotein foci must also be considered. Many important questions remain to be answered; indeed, we are just beginning to understand translational regulation in plants and can thus anticipate major findings in this field in coming years.

## Author Contributions

All authors listed made substantial intellectual contribution to the work, wrote the manuscript together and approved it for publication.

## Conflict of Interest Statement

The authors declare that the research was conducted in the absence of any commercial or financial relationships that could be construed as a potential conflict of interest.
